# Conformational activation and disulfide exchange in HIV-1 Env induce cell-free lytic/fusogenic transformation and enhance infection

**DOI:** 10.1128/jvi.01471-24

**Published:** 2025-02-06

**Authors:** Charles G. Ang, Nadia L. Hyatt, Giang Le Minh, Monisha Gupta, Manali Kadam, Philip J. Hogg, Amos B. Smith, Irwin M. Chaiken

**Affiliations:** 1 Department of Biochemistry and Molecular Biology, College of Medicine, Drexel University 427309 , Philadelphia, Pennsylvania, USA; 2 Department of Chemistry, College of Arts and Sciences, Drexel University 171623 , Philadelphia, Pennsylvania, USA; 3 School of Life Sciences, University of Technology Sydney and Centenary Institute, The University of Sydney 4334 , Sydney, New South Wales, Australia; 4 Department of Chemistry, University of Pennsylvania 312071 , Philadelphia, Pennsylvania, USA; Icahn School of Medicine at Mount Sinai, New York, New York, USA

**Keywords:** HIV-1, Env, thioredoxins, allosteric disulfides, virus entry, entry inhibitors, thiols, disulfide exchange

## Abstract

Disulfide exchange is underexplored as a mechanism influencing HIV-1 entry. Prior studies demonstrated that redox enzyme inhibition can prevent HIV-1 infection but with limited mechanistic explanation. We hypothesize that ligand-driven rearrangement (“conformational activation”) enables enzyme-mediated disulfide exchange in Env residues (“disulfide trigger”) that promotes fusion transformations, enhancing virus entry. We tested soluble CD4 and CD4-binding site entry inhibitors as conformational activators and the ubiquitous redox enzyme thioredoxin-1 (Trx1) as disulfide trigger. We found that combination treatment caused fusion-like Env transformation and pseudovirus lysis, independent of cells. Notably, only compounds associated with gp120 shedding caused lysis when paired with Trx1. In each case, lysis was prevented by adding the fusion inhibitor T20, demonstrating that six-helix bundle formation is required as in virus–cell fusion. In contrast to conformationally activating ligands, neither the ground state stabilizer BMS-806 with Trx1 nor Trx1 alone caused lysis. Order of addition experiments reinforced conformational activation/disulfide trigger as a sequential process, with virus/activator preincubation transiently enhancing lysis and virus/Trx1 preincubation reducing lysis. Lastly, addition of exogenous Trx1 to typical pseudovirus infections exhibited dose-dependent enhancement of infection. Altogether, these data support conformational activation and disulfide triggering as a mechanism that can induce and enhance the fusogenic transformation of Env.

**IMPORTANCE:**

HIV remains a global epidemic despite effective anti-retroviral therapies (ART) that suppress viral replication. Damage from early-stage infection and immune cell depletion lingers, as ART enables only partial immune system recovery, making prevention of initial virus entry preferable. In this study, we investigate disulfide exchange and its facilitating conformational rearrangements as underexplored, but critical, events in the HIV entry process. The HIV envelope (Env) protein effects cell entry by conformational rearrangement and pore formation upon interaction with immune cell surface proteins, but this transformation can be induced by Env’s conformational activation and disulfide exchange by redox enzymes, which then integrates into established processes of HIV entry. The significance of this research is in identifying Env’s conformational activation as a mechanistic requirement for initiating fusion by triggering disulfide exchange. This will aid the development of novel preventative strategies against HIV entry, particularly in the context of HIV-enhanced inflammation and comorbidities with redox mechanisms.

## INTRODUCTION

Research into HIV-1 entry has largely focused on three key proteins, HIV-1 Env, host cell CD4, and host cell CCR5 or CXCR4, and successfully developed a functioning mechanistic understanding of virus–cell fusion (1). However, additional mechanisms and interactions may still underlie and support the established model of fusion and could be exploited to develop novel interventions and treatments. Disulfide bonds between cysteine residues usually link and stabilize protein tertiary and quaternary structures to form their native states, but other disulfides have strained configurations that can significantly impact a protein’s functionality when forming or breaking, which have been termed as allosteric disulfides (2–4). HIV-1 Env natively contains nine disulfides in its gp120 subunit and one in its gp41 subunit (5, 6), and bond angle and functional analyses have identified gp120 cysteine pairs at positions C126–C196, C296–C331, and C385–C418 as potential allosteric disulfide pairs (4, 7, 8), which may be targeted by redox enzymes, such as thioredoxin-1 (Trx1) and protein disulfide isomerase (PDI, PDIA1) (8).

Targeted Trx1 and PDIA1 inhibition has been found to suppress HIV-1 infection *in vitro* (7, 9–14), suggesting that disulfide exchanges are key contributors to virus–cell infection. Thioredoxin family enzymes are found in virtually all organisms at intra- and extra-cellular locations and contribute to a host of functions, including proper protein folding, modulating extracellular clotting factors, and localization of surface proteins, including CD4 (14–21). Lastly, a C35S Trx1 trapping mutant was used against Env gp120 to identify the initial gp120-Trx1 mixed disulfide as C296–C331, indicating the probable target of Trx1’s initial interactions with Env (22).

The Env C296–C331 disulfide also has been found to be targeted by peptide triazole thiols (PTTs). Peptide triazoles (PTs) are a class of HIV-1 entry inhibitors that target Env, functioning by filling Env’s CD4-binding pocket with its isoleucine–triazoleproline–tryptophan (IXW) pharmacophore and distorting Env’s coreceptor binding site, resulting in shedding of Env’s gp120 subunit and irreversible inactivation of the virus (23–26). PTTs are a PT variation featuring an additional free thiol *via* terminal cysteine (27, 28). PTTs not only retain the ability to cause irreversible inactivation via shedding, but also induce further transformation of Env, resulting in six-helix bundle (6HB) formation and membrane disruption, lysing virus particles, and porating Env-presenting cells (28, 29).

**Fig 1:**
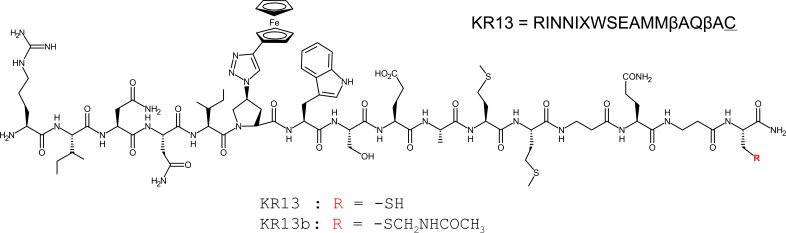
Structure and amino acid sequence of the PTT KR13 and its thiol-blocked analogue KR13b, which functions as a PT.

The mechanistic role of the PTT terminal cysteine was examined by comparing the PTT KR13 with a modified variant that contains a protective acetamidomethyl group to block the free thiol on cysteine, denoted as KR13b (Fig. 1) (28, 29). KR13b functions as a non-thiol PT capable of causing gp120 shedding, irreversible inactivation, and HIV-1 infection inhibition but not virus lysis or membrane disruption (28). Docking and mutational experiments showed that only the C296–C331 disulfide pair was both within KR13’s reach when docked at the CD4-binding site and required to be intact for virus lysis, demonstrating reliance on disulfide exchange (30).

We hypothesize that since PTT targets the C296–C331 allosteric disulfide of Env, PTT is accessing a native capacity of Env normally mediated by surface-associated or extracellular Trx1 after initial virus–cell encounter and CD4 engagement, where it would induce membrane disruption to facilitate cell entry and infection. This would separate PTT functionality into a pharmacophore-mediated conformational activation paired with a thiol-mediated disulfide trigger to achieve Env transformation and membrane disruption. We investigated if conformational activation is the mechanistically key structural element needed to prepare Env for a disulfide trigger by examining other Env-targeting inhibitors as activator surrogates.

## MATERIALS AND METHODS

### Cell culture

HEK293T cells (CRL-3216) were obtained from ATCC (Manassas, VA, USA). The following reagent was obtained through the NIH HIV Reagent Program, Division of AIDS, NIAID, NIH: HOS CD4+ CCR5+ cells, ARP-3318 contributed by Dr. Nathaniel Landau, Aaron Diamond AIDS Research Center, The Rockefeller University.

### Plasmids

JRFL wild-type Env was a kind gift from Dr. Simon Cocklin. HXB2 wild-type Env was a kind gift from Dr. Joseph Sodroski. NL4-3.R^-^E^-^.LucAM was a kind gift from Dr. Nathan Landau. The following reagent was obtained through the NIH HIV Reagent Program, Division of AIDS, NIAID, NIH: human immunodeficiency virus 1 (HIV-1) BaL.01 Env expression vector, ARP-11445 contributed by Dr. John Mascola. The following reagent was obtained through the NIH HIV Reagent Program, Division of AIDS, NIAID, NIH: plasmid pHEF expressing vesicular stomatitis virus (VSV-G), ARP-4693, contributed by Dr. Lung-Ji Chang.

### Pseudovirus production

Pseudotyped JRFL, Bal.01, HXB2, and VSV viruses were produced by transient transfection of HEK293T cells (3 million cells in T75 tissue culture flasks) with plasmids encoding surface spike (either Env or VSV-G, 4 µg DNA) and NL4-3.R^-^E^-^.LucAM (8 µg DNA) using 1 mg/mL PEI as described previously (28, 29, 31). Supernatants were collected at 72 h after transfection, gradient purified, and quantified for p24 by enzyme-linked immunosorbent assay (ELISA). Purified pseudovirus was also validated for infectivity against HOS CD4+ CCR5+ cells by luminescence assay as described previously (28, 29, 31). HOS CD4+ CCR5+ cells are permissive for CXCR4 expression and can allow for productive X4-tropic infection (32).

### PT and cPT production and purification

All PTs and cPTs reported in this paper were synthesized via solid-phase peptide synthesis using the Liberty Blue microwave peptide synthesizer (CEM, Matthews, NC) and other processes, such as click conjugation as previously reported (28, 33, 34).

All reagents used in synthesis were purchased from vendors, such as CHEM-IMPEX, Alfa Aesar, Ambeed, and Sigma Aldrich, and of high-performance liquid chromatography (HPLC) grade unless noted otherwise. HPLC purifications were conducted with a Waters Semi-preparative HPLC System. Purity and mass verification of synthesized peptides were performed with a Waters Analytical HPLC System equipped with the Acquity QDa. All compounds were verified to greater than or equal to 95% homogeneity; yields were calculated based on the amount of purified product obtained.

### Recombinant human thioredoxin-1

Trx1 was purchased from Cayman Chemical (Ann Arbor, MI).

### Small molecule CD4 mimetic production

The small-molecule CD4 mimetics BNM-III-170 and CJF-III-288 were produced as previously reported (Refs. [35] and [36], respectively).

### Soluble CD4 production

Soluble CD4 (sCD4) was produced recombinantly as described previously (26, 37). Briefly, CHO-ST4.2 cells, which secrete the full extracellular domain of CD4, were grown in a hollow-fiber bioreactor (Fiber-Cell Systems, Inc.). The supernatant was collected and fractionated over a sulfopropyl-substituted ion-exchange column (GE Healthcare), followed by a quaternary ammonium-substituted ion-exchange column (GE Healthcare) with an AKTA fast protein liquid chromatography instrument (GE Healthcare). The column flow-through containing purified CD4 was pooled and dialyzed into 1× phosphate-buffered saline (PBS) (pH 7.4) overnight at 4°C. All protein preparations were analyzed by sodium dodecyl sulfate-polyacrylamide gel electrophoresis (SDS-PAGE) and Coomassie staining (Invitrogen) and found to be >95% homogeneous.

### ELISA for p24 detection

ELISA plates were first prepared by incubating 50 µL per well of 1 ng/µL mouse monoclonal anti-p24 (Abcam) in phosphate-buffered saline (1× PBS) overnight at 4°C, then blocked for 2 h with 3% bovine serum albumin and 0.1% Tween 20 in PBS. Fixed quantities of JRFL pseudovirus (250 ng p24 worth) were incubated with serial dilutions of PTT, PT, soluble CD4, small-molecule CD4 mimetics, and/or recombinant human Trx1 at 37°C for 2 h before spinning at 15,000×*g* for 2 h to pellet any virus debris and intact virus. Supernatants were then collected and added in quadruplicate to the prepared and blocked 96-well ELISA plates for analysis. Controls used were PBS treatment of pseudovirus (negative) and 1% Triton-X in PBS treatment of pseudovirus (positive). After incubation overnight at 4°C, plates were emptied of sample, washed once with 0.1% Tween 20 in PBS, then incubated for 1 h at room temperature on a shaker with 1:3,000 rabbit polyclonal anti-p24 (Abcam) in 0.5% BSA in PBS as primary. After primary incubation, plates were washed thrice with 0.1% Tween 20 in PBS, then incubated for 1 h at room temperature on a shaker with 1:3,000 donkey polyclonal anti-rabbit IgG linked to horseradish peroxidase (Abcam) in 0.5% BSA in PBS as secondary. After secondary incubation, plates were washed thrice with 0.1% Tween 20 in PBS and once with PBS before light-protected incubation for 30 min at room temperature on a shaker with 180 µL of 0.4 mg/mL O-phenylenediamine (Sigma) in phosphate–citrate buffer with sodium perborate (Sigma). After development, plates were read on a Tecan Infinite F50 plate reader and processed with GraphPad Prism 9 for analysis, statistics, and data plotting.

### Pseudovirus cell infection assay

JRFL, BaL.01, HXB2, or VSV pseudovirus (50 ng p24 per well) and serial dilutions of Trx1 (starting from 1,000 nM and diluting by four each time) were simultaneously added to 96-well plates containing HOS CD4+ CCR5+ cells (10,000 cells per well, plated 24 h prior to infection). After infection, plates were incubated for 24 h, and then their media were removed and replaced with fresh media. After media change, plates were incubated for another 24 h, and then their cells were lysed with 1× passive lysis buffer and measured for infectivity by luminescence assay as described previously (28, 29, 31).

## RESULTS

### Establishing conformational activation and disulfide exchange trigger as a functional combination

Our previous work on the comparative transformations elicited by peptide triazoles and peptide triazole thiols (29) demonstrated that the only difference between lytic and non-lytic transformations of Env by KR13 and its thiol-protected variant KR13b was the availability of the terminal cysteine’s –SH. Here, we propose that this free thiol may mimic the functionality of native redox enzymes that cause disulfide exchange in the normal course of HIV-1 infection (11–14), and that the IXW pharmacophore acts as an activating component to Env, inducing a temporary activated state that can then be triggered for further conformational rearrangement by disulfide exchange.

To examine the view of PTT as a combination of activator pharmacophore and thiol trigger, we tested if a combination of PT plus recombinant human thioredoxin-1 (Trx1) could recapitulate the lytic transformation effect of PTT in a cell-free environment (and, thus, free of endogenously produced Trx1).

**Fig 2:**
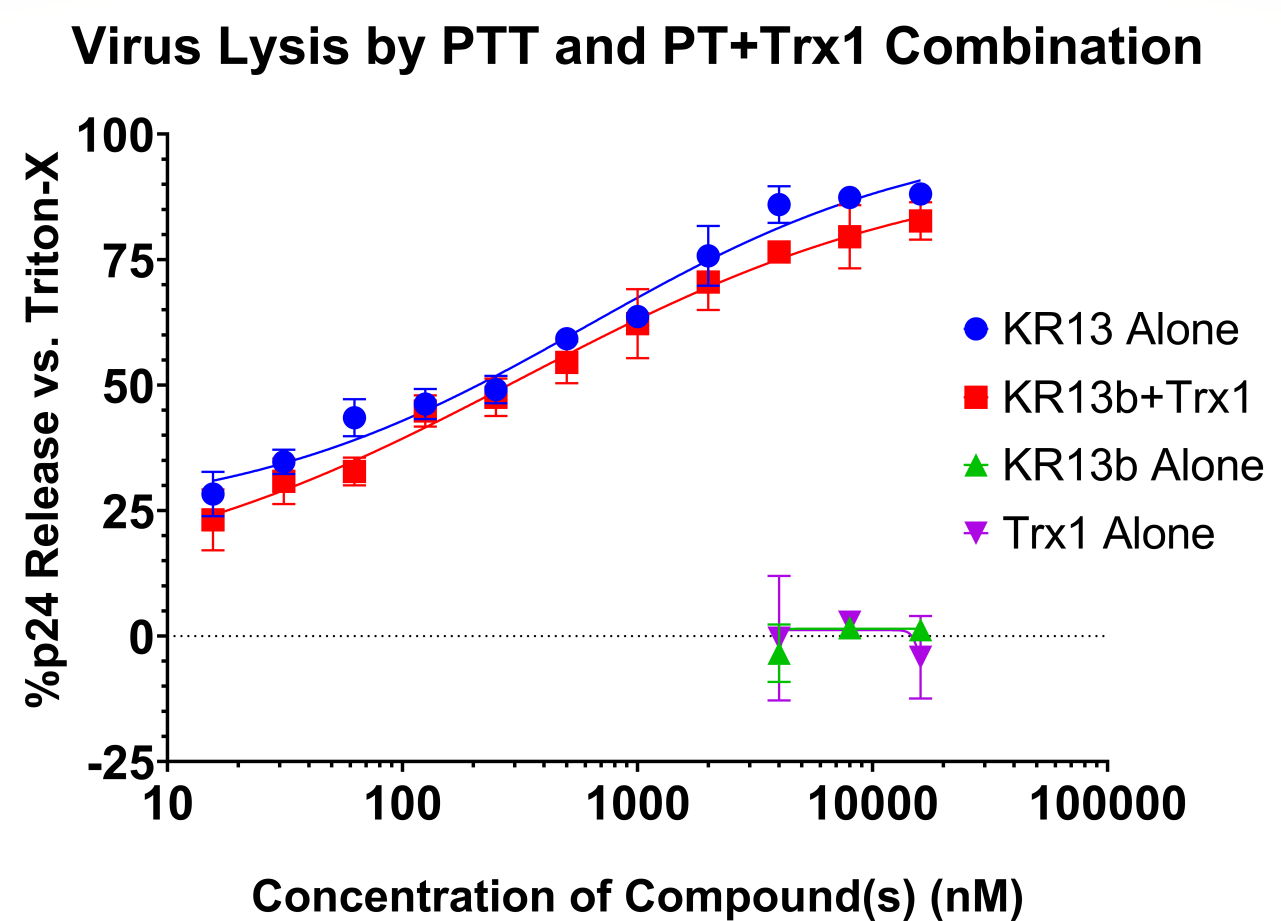
Dose-dependent lysis of JRFL pseudovirus by treatment with either PTT KR13 or a combination of PT KR13b and recombinant human thioredoxin-1. Molar ratio of 1:1 was maintained for the KR13b + thioredoxin-1 dilutions. Controls of KR13b alone and Trx1 alone showed no significant p24 release. Data are presented as the mean of three independent experiments, and error bars show the standard deviation. Calculated EC_50_ values are 609 ± 184 nM for KR13 alone and 317 ± 158 for the combination of KR13b and Trx1 together. Data are presented as the mean of three independent experiments, and error bars show the standard deviation.

Because the PTT KR13 contains the IXW pharmacophore and the unmodified thiol, we used a 1:1 molar ratio of PT KR13b (thiol-blocked KR13, Fig. 1) and Trx1 for this experiment. For the membrane disruption and p24 leakage effect, the combination of PT + Trx1 was shown to recapitulate the capabilities of PTT alone (Fig. 2), suggesting that the PTT’s thiol and redox enzymes are performing the same function when in the presence of the IXW pharmacophore. We further tested whether the addition of Trx1 altered the profile of KR13b-induced gp120 shedding and found that it did not (Fig. S1), suggesting that shedding is a shared event, regardless of the eventual lytic fate of the virus.

**Fig 3:**
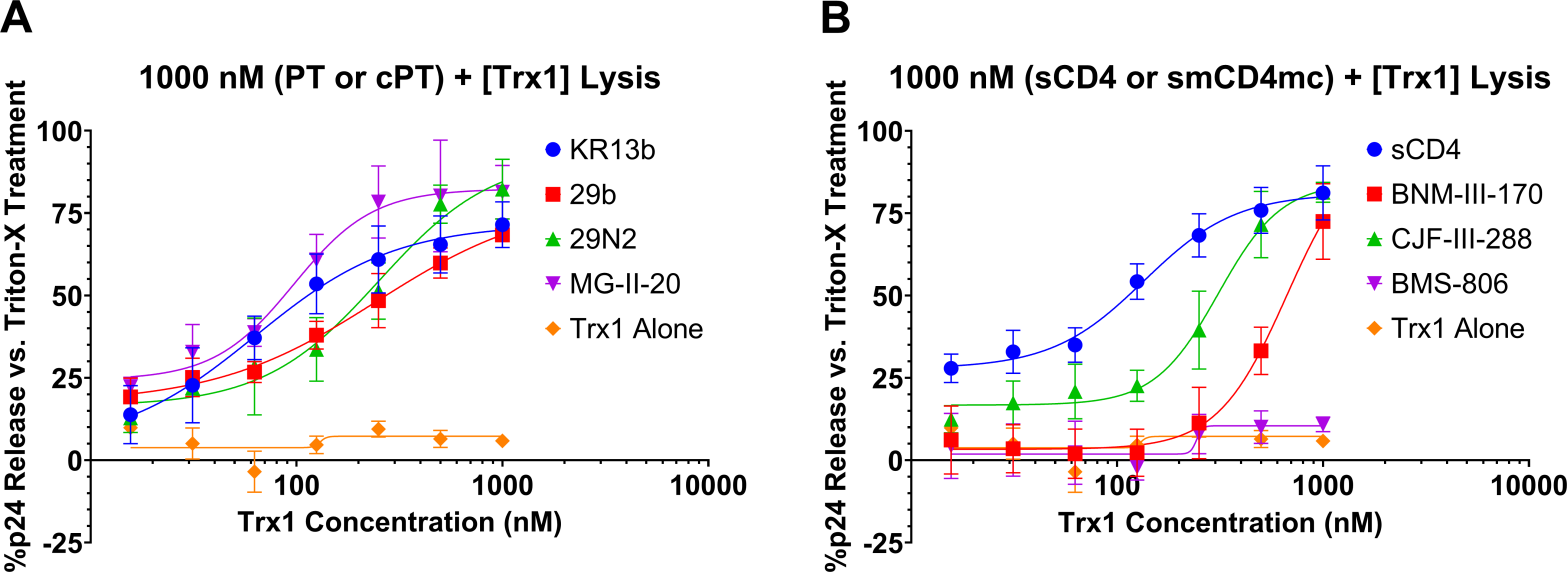
(A) Dose-dependent lysis of JRFL pseudovirus by treatment with fixed 1,000 nM KR13b, 29b, 29N2, or MG-II-20 combined with serial dilutions of thioredoxin-1. (B) Dose-dependent lysis of JRFL pseudovirus by treatment with fixed 1,000 nM sCD4, BNM-III-170, CJF-III-288, or BMS-806 combined with serial dilutions of thioredoxin-1. Data presented are the mean of three independent experiments, and error bars show the standard deviation. EC_50_ values are summarized in Table 1.

We next examined the dose dependence of Trx1 on the lysis effect. The concentration of the conformational activating component was held constant (1,000 nM) while serially diluting the concentration of Trx1 (starting from 1,000 nM with 1/2 serial dilutions). The data for these analyses are given in Fig. 3.

**TABLE 1:** EC_50_ values for lysis of JRFL pseudovirus by 1,000 nM entry inhibitor + dilutions of Trx1 derived from Fig. 3^*a*^

	KR13b	29b	29N2	MG-II-20	sCD4	BNM-III-170	CJF-III-288	BMS-806	Trx1 alone
EC_50_ (nM)	63 ± 25	246 ± 97	251 ± 84	97 ± 18	135 ± 21	666 ± 315	309 ± 44	–^*b*^	–^*b*^
K_d_ (nM)	6.13 (SPR) (28)	415 ± 98 (ITC) (34)	350 ± 62 (ITC) (34) 107 ± 4 (SPR) (38)	105.2 ± 1.2 (SPR) (38)	22 ± 6 (SPR) (39)	47 (ITC) (35)	3.10 ± 0.6 (SPR)^*c*^	21.1 ± 1.9 (SPA) (40)	–^*d*^

^*a*^ For comparison, gp120 binding affinities (and method) are shown as determined previously when available (SPR: surface plasmon resonance; ITC: isothermal titration calorimetry; SPA: scintillation proximity assay).

^*b*^ Lysis was not observed with this compound.

^*c*^ Data provided by personal correspondence from Dr. Gabriela Canziani.

^*d*^ Binding of Trx1 to HIV-1 Env alone was not performed or found in literature.

The IXW pharmacophore of PTs and cPTs used in the analyses competes for the CD4-binding site, inserting into Env’s Phe43 pocket and distorting Env’s coreceptor binding site (23, 33). PTs and cPTs provide engagement with Env analogous to soluble CD4 (sCD4), which agrees with observations of gp120 shedding caused by sCD4, PTs, and cPTs (28, 33, 41, 42).

Besides KR13b, we tested its cyclized variant, AAR029b (29b), which features the same ferrocene extension on the triazoleproline as KR13 and KR13b (33). We also tested the second-generation cPT AAR029N2 (29N2), which replaces ferrocene with a phenyl–thiophene (34), and the third-generation cPT MG-II-20, which uses a phenyl–pyrazole in the same location (38). Structures for AAR029b, AAR029N2, and MG-II-20 are provided in Fig. S2.

For further generalization of the conformational activation/disulfide trigger hypothesis, we tested the combinations of recombinant Trx1 with soluble CD4 itself, as well as small-molecule CD4 mimetics, and an Env conformational blocker. BNM-III-170 is a small-molecule CD4 mimetic shown to be inactivating at full Env trimer occupancy but transiently activating at lower occupancies, potentially leading to infection in CD4-negative/CCR5-positive cell lines (43). CJF-III-288 is an indoline-based evolution of the BNM-III-170 small-molecule CD4 mimetic that has been shown to have enhanced antiviral potency (36). In contrast, BMS-378806 (BMS-806) is known to be a small-molecule inhibitor of Env (and developmental precursor to Temsavir) binding in the CD4 pocket and stabilizing the ground state of Env to prevent conformational changes (43–45). Dilutions of thioredoxin-1 alone were also tested as a control for any non-activated lysis. Structures for BNM-III-170, CJF-III-288, and BMS-806 are provided in Fig. S3.

As in the PTT vs. PT + Trx1 assay (Fig. 2), combining KR13b with Trx1 resulted in p24 release. However, by holding the KR13b concentration steady, we specifically observed the dose dependence of lysis on the Trx1 concentration (Fig. 3A). Substituting the cyclic PT compounds for the linear, we still observed dose-dependent lysis by Trx1 with all three cPTs, albeit at higher EC_50_ values (Fig. 3A, Table 1). Nonetheless, the preserved lytic effect demonstrated that Trx1-triggered transformations are not limited to the linear peptide triazoles and peptide triazole thiols, showing a viable path for development of cyclic peptide triazole thiols (cPTTs).

Lysis by sCD4 + Trx1 allows us to further generalize the activator-and-trigger hypothesis and creates further links to the events and transformations of native fusion (Fig. 3B). While BNM-III-170 and CJF-III-288 did cause lysis in conjunction with Trx1, notably, BMS-806 did not (Fig. 3B, Table 1). This further demonstrated that simple occupancy of the CD4 binding site is insufficient to allow for transformational trigger by disulfide exchange, and that an activating rearrangement is necessary. Furthermore, treatment of viruses with Trx1 alone in the absence of any activating component showed negligible lysis, indicating that it alone is insufficient to trigger rearrangement.

As an additional combinatorial experiment, we treated JRFL pseudovirus with mixtures of 1,000 nM KR13b and 1,000 nM of either free cysteine or glutathione. Neither thiol compound caused leakage of p24 above background (Fig. S4).

### Order of addition

We next performed an order-of-addition experiment to confirm that virus lysis was a two-step process: conformational activation of Env by the inhibitor, followed by triggering of Env rearrangements by Trx1. Prior data on sCD4 and CD4 mimetics reported that they induce a short-lived activated state (defined by availability of the HR1 groove) during inhibition of the Env trimer (42), supporting a two step process.

**Fig 4:**
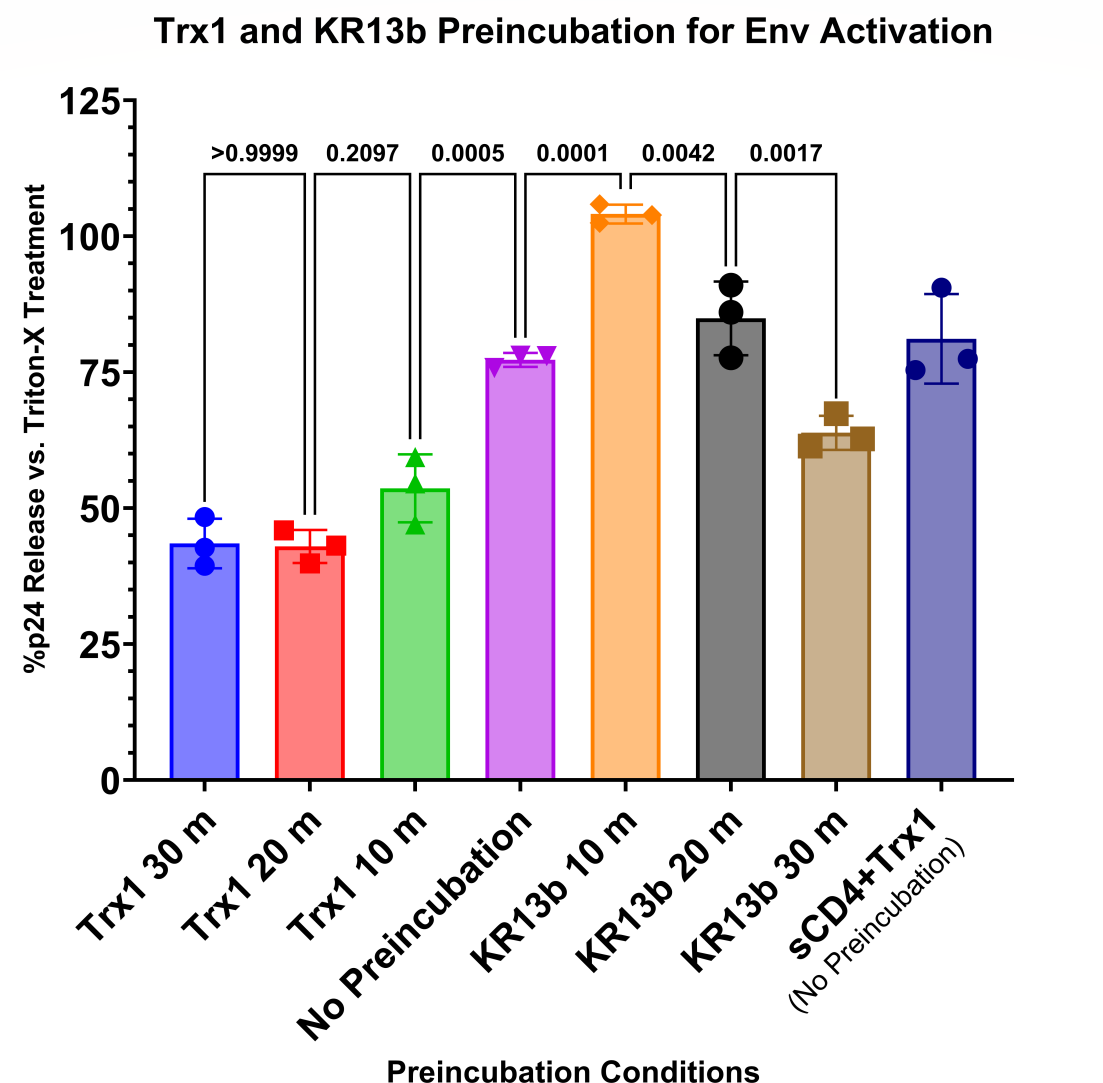
Preincubation of JRFL pseudovirus with Trx1 or KR13b influences efficacy of lysis. Pseudovirus was preincubated with either 1,000 nM KR13b or 1,000 nM Trx1 for 0–30 min before addition of the complementary component, then incubation for 2 h at 37°C. A control of sCD4 + Trx1 treatment of pseudovirus (1,000 nM each, no preincubation) is included for comparison. Data are presented as the mean of three independent experiments, and error bars show the standard deviation. Significances of differences between preincubation conditions are listed as *p*-values above adjacent bars and calculated by one-way analysis of variance with Tukey’s multiple comparisons test, with a single pooled variance.

Virus was preincubated with 1,000 nM KR13b or Trx1 for 0–30 min before addition of the remaining component (1,000 nM Trx1 or KR13b, respectively, or both for the no preincubation sample) and further incubation for 2 h at 37°C. Released p24 was then quantified by ELISA to determine virus lysis and leakage (Fig. 4). Notably, preincubation with the “activating” component of KR13b resulted in a significantly increased lysis at 10 min preincubation but dropped off with longer preincubation to less lysis at 30 min preincubation than the sample with no preincubation. In contrast, preincubation with Trx1 showed a significant decrease in lytic effect at 10 min and decreased further through 20 min of preincubation. For the KR13b preincubation, lysis peaking at 10 min would align with transient activation by sCD4 and CD4 mimetics, assuming all of these activated states are more conducive to transformation down Env’s native fusion pathway (42, 43).

### Exogenous Trx1 increases pseudovirus infection

To validate the possibility of Trx1 interacting with the native fusion process, we conducted a straightforward pseudovirus infection assay of JRFL Env, BaL.01 Env, HXB2 Env, and vesicular stomatitis virus (VSV) pseudovirus with HOS CD4+ CCR5+ target cells supplemented with serial dilutions of Trx1. VSV was included as a control, as it uses a non-Env-dependent entry mechanism. Pseudovirus and Trx1 were combined at room temperature and added to cells with no pre-incubation to minimize any premature pseudovirus–Trx1 or cell–Trx1 interactions.

**Fig 5:**
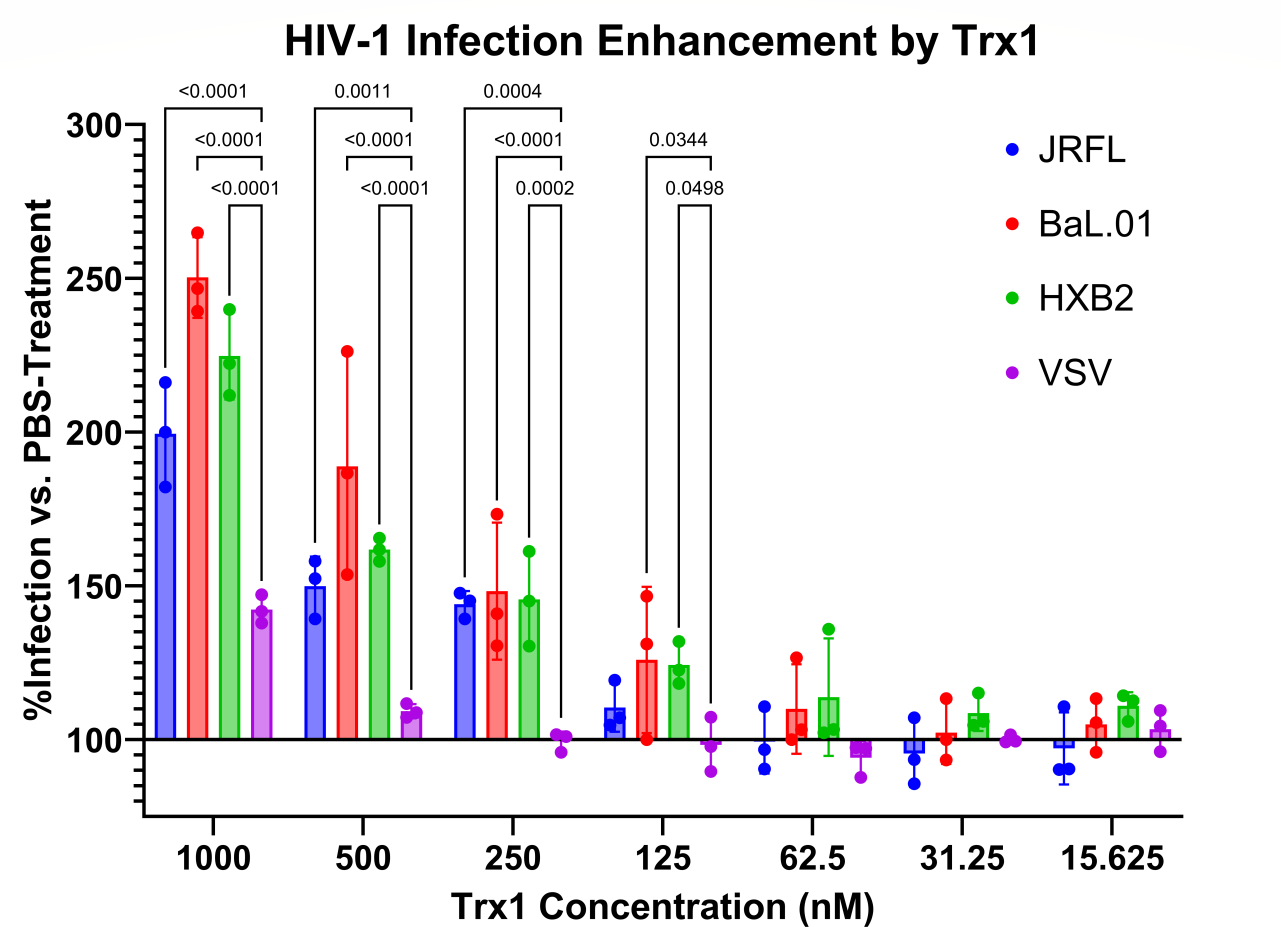
Trx1 dose-dependent increase of JRFL, BaL.01, and HXB2 pseudovirus infection. VSV pseudotyped virus was included as a non-Env control. HOS CD4+ CCR5+ cells (CXCR4 permissive) were infected with pseudoviruses supplemented with serial dilutions of Trx1. Infection levels for each pseudotype were normalized against a pseudovirus + PBS (solvent) control. Experiment was performed in quadruplicate wells. Data are presented as the mean of three independent experiments, and error bars show the standard deviation. Significances of differences between Env subtypes and the VSV control are listed as *p*-values above adjacent bars and calculated by one-way analysis of variance with Tukey’s multiple comparisons test, with a single pooled variance.

Supplementing the infection assays with Trx1 enhanced the infectivity of the pseudovirus (Fig. 5), at least doubling its RLU values at 1,000 nM of Trx1. Although high concentrations (500–1,000 nM Trx1) enhanced VSV infection (~10–40%), the infection increase observed in every Env pseudovirus significantly exceeded the VSV increase.

### T20 inhibition of lytic effect

The lytic effect of the PTT KR13 has previously been shown to be inhibited by the fusion inhibitor T20/Enfuvirtide (28), which itself is a 36-amino acid peptide derived from gp41’s C-terminal heptad repeat (CHR) region and functions by lodging itself in the grooves of trimerized gp41 N-terminal heptad repeats, sterically preventing gp41’s own CHRs from folding in during 6HB formation in native fusion (46). This was further validated by flow cytometric analysis demonstrating increased binding of the 6HB-specific antibody NC-1 after KR13 treatment of cell surface-expressed Env and inhibition of NC-1 binding by co-treatment with KR13 and T20 together (29). T20 inhibition suggested that KR13 functioned upstream of formation of the pre-hairpin intermediate and subsequent refolding into the 6HB, interacting primarily with Env gp120’s CD4-binding, coreceptor-binding, and C296–C331 sites.

To demonstrate commonality between the combination of conformational activator and disulfide exchange trigger in this study, the action of single-molecule PTT, and native fusion, we reprised the T20 inhibition of lysis experiment with a simultaneously added mixture of 1,000 nM KR13b, 1,000 nM Trx1, and 0–1,000 nM T20 to JRFL pseudovirus, incubation for 2 h at 37°C, and detection of released p24 by ELISA.

**Fig 6:**
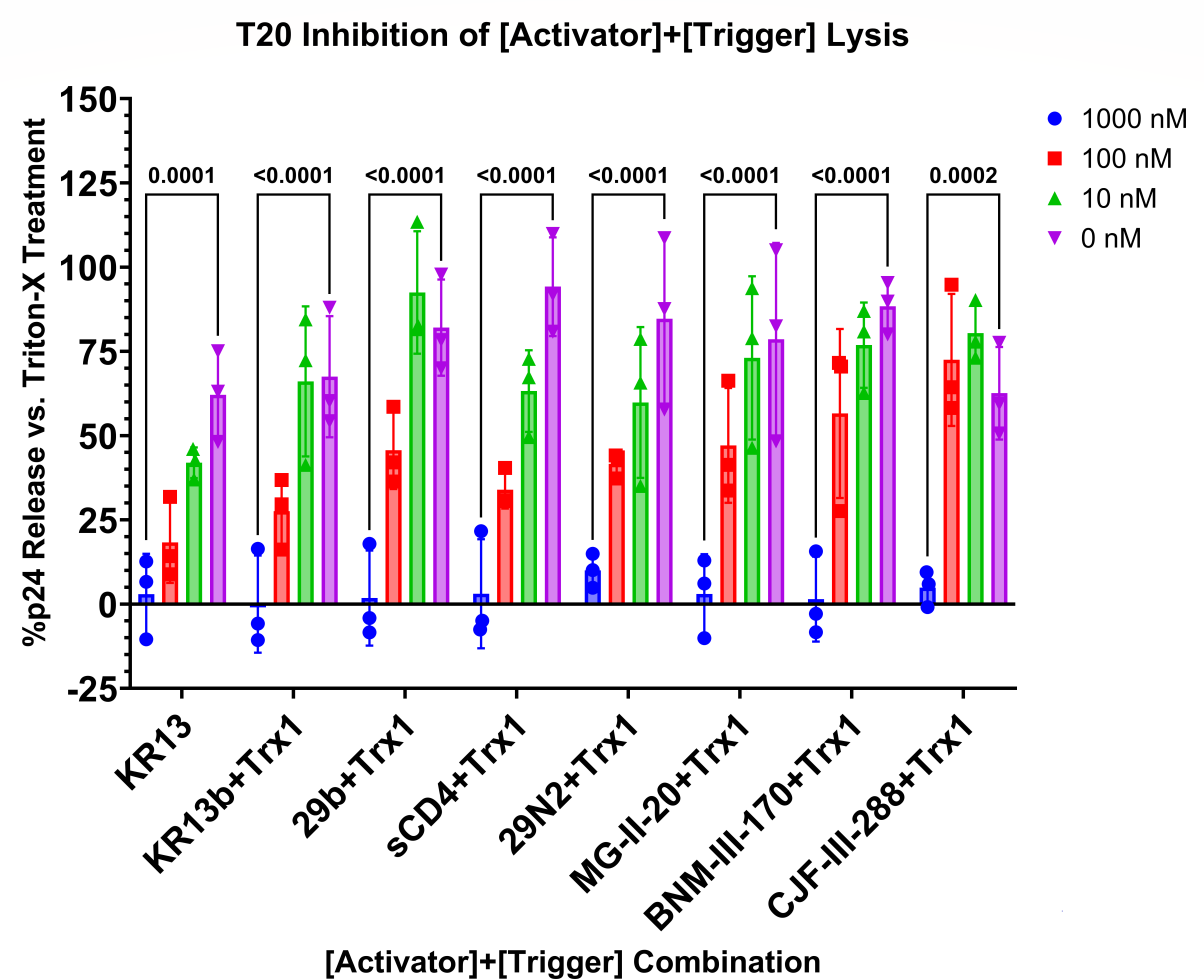
T20 inhibition of KR13b + Trx1-induced lysis of JRFL pseudovirus. Pseudovirus was co-incubated with 1,000 nM KR13b, 1,000 nM Trx1, and 0–1,000 nM T20 for 2 h at 37°C before evaluation of released p24 by ELISA. Data are presented as the mean of three independent experiments, and error bars show the standard deviation. Significances of differences between 1,000 nM T20 concentrations and untreated (0 nM) controls are listed as *p*-values above bars and calculated by two-way analysis of variance with Tukey’s multiple comparisons test, with a single pooled variance.

The addition of T20 prevented lysis of pseudoviruses by each combination of conformational activator and Trx1 (Fig. 6), confirming that 6HB is a required downstream element of this two-component lysis effect, just as it is with the single component PTT KR13, and native fusion. These data reinforce the view that the conformational activation/disulfide exchange process and the Env conformational changes occurring during cell infection are related.

## DISCUSSION

### HIV-1 Env disulfide/redox enzyme interactions can be exploited for fusion-like transformation

The data presented in this work support the overall hypothesis of HIV-1 Env being susceptible to a conformational activation/disulfide trigger mechanism to induce the lytic transformation of the Env trimer and further link these transformations to Env’s native fusogenic transformations.

The Env-driven mechanism of virus–cell entry as is understood (1) describes significant structural changes in Env that are needed to achieve the function of fusion, and disulfide bonds could be a major passive and active component of those changes. Several previous reports have investigated the role of disulfides and native redox enzymes in the Env-mediated infection process, including modulating CD4’s D2 structure and membrane localization (7, 9, 14) and interactions with the Env/CD4–coreceptor complex (12, 47–49), while their targeted inhibition can prevent successful infection by the virus (7, 13, 48). In Env, Barbouche et al. found an overall reduction of two disulfide bonds in gp120 after encounter with CD4^+^ T cell surfaces, but whether this resulted directly from two reductions or was the net result after a series or cascade of disulfide exchanges was not clear (50). A study by van Anken et al. disabled each disulfide pair in Env by mutation and tracked how far the resulting protein could go in the viral lifecycle: C119–C205 and C378–C445 could be disabled with no penalty to fusion; C385–C418 mutations caused severe folding defects but allowed some residual fusion function; and the remaining seven pairs were required for fusion (C54–C74, C126–C196, C131–C157, C218–C247, C228–C239, C296–C331, C598–C604) (6). CD4’s domain two disulfide bond (C130–C159) is uniquely strained and unstable compared to its domain 1 and 4 disulfides, and reduction of C130–C159 enables CD4 dimerization via domain swapping (C130 of the first monomer bonds to C159 of the second monomer, and vice versa) (9, 51, 52). Dimerization allows CD4 to bind Class II MHC to perform its immune functions (52). In contrast, HIV Env preferentially interacts with reduced monomeric CD4 to effect entry (53), but Env binding also impairs subsequent CD4 dimerization (54) potentially dampening immune function.

Most commonly implicated in HIV-1 infection are the enzymes thioredoxin-1 (Trx1, TXN) and protein disulfide isomerase (PDI, PDIA1, P4HB), which share an enzymatic (thioredoxin-family) motif and many targets, initiating disulfide exchanges to modulate protein conformation (8). While structural disulfides contribute to defining tertiary and quaternary protein structures and enzymatic disulfides catalyze other disulfide exchanges, allosteric disulfides modulate mature protein activity when formed or broken (2–4, 55). In the context of HIV-1 Env, C126–C196, C296–C331, and C385–C418 have been identified as potential allosteric disulfides in Env’s gp120 subunit (4), and Trx1 specifically and preferentially targets the C296–C331 disulfide (22). Notably, the C296–C331 disulfide has the same strained –RHstaple conformation as the allosteric disulfide bond cleaved in CD4 (3).

Prior work by Bailey et al. (30) found that of the disulfide bonds of Env plausibly accessible to PTT with its pharmacophore located at the CD4-binding site (C296–C331, C375–C445, C385–C415), only mutation of C296–C331 disabled lysis, suggesting that the other two are not involved in either initial binding or any exchange cascade. Bailey additionally found that mutating the C598–C604 pair reduced KR13-induced lysis, suggesting the possibility that C598–C604 could be part of a disulfide exchange cascade, even if not a direct target of KR13.

For Trx1 to trigger disulfide exchange in Env and start lytic/fusogenic rearrangements, we observed that Env needed to interact with other ligands first, presumably to prime Env by inducing a needed conformation. We were able to do this by treating the virus with “conformational activator” Env-targeting entry inhibitors, here including PT, cPT, small-molecule CD4 mimetics, and soluble CD4 itself, all of which are known to induce conformational change in Env (Fig. 2, 3A and B). In contrast, a ground-state stabilizing Env binder, BMS-806, was an “inactivating” entry inhibitor, and the lack of an activator resulted in no Trx1-triggered lysis.

Based on the closed Env SOSIP.664 structure 5VN8 (56), the location of the C296–C331 bond of gp120 at the base of the V3 loop is relatively exposed, and Trx1 can access it as the Trx1 preincubation experiment demonstrated without prior conformational change (Fig. 4). This does not discount the possibility of PT/PTT binding to further expose C296–C331, to position other disulfides for exchange cascades, or to otherwise reconfigure Env to promote lysis/fusion, but production of a PT-bound Env structure that would reveal this is still underway.

PT/PTT treatment does induce known conformational changes in Env, though not identical to those from CD4. Binding of mAb 17b, a coreceptor surrogate, is heavily suppressed after both PT and PTT engagement (23, 28, 30). PTT treatment reveals MPER epitopes (10E8) but seems to decrease access to the immunodominant loop (50-69) and the fusion peptide (VRC34.01) in forming its six-helix bundle (NC-1) (29). PT treatment instead increases access to the immunodominant loop and fusion peptide, with no change in MPER and no six-helix bundle formation (29). Expanding these experiments into a full antibody panel combined with molecular modeling of the post-PT/PTT-treated Env spike is a goal we are pursuing.

In the Trx1 supplementation experiments (Fig. 5), we found substantial increases in infection across all Env subtypes tested. JRFL (R5-tropic) is considered Tier 2 for neutralization; BaL.01 (R5-tropic) and HXB2 (X4-tropic) are considered Tier 1B; and VSV is the pseudovirus with Env replaced by the G protein of vesicular stomatitis virus utilizing LDLR-mediated endocytosis for entry. Trx1 treatment of VSV during infection had minimal effect, except at very high concentrations, increasing signal by ~10% at 500 nM and ~40% at 1,000 nM, suggesting either a general enhancement of protein production or endocytosis. Importantly, effects on JRFL, BaL.01, and HXB2 infection all exceeded the observed effects on VSV and with possibly greater infection enhancement in the Tier 1B BaL.01 and HXB2. Greater likelihood of destabilization in low-neutralization tier Env trimers would lead to greater infection in a permissive environment exhibited by the Trx1 addition experiment. This also suggests that Trx1 or similar redox enzymes may be a limiting reagent in infection, although perhaps explained by the relative abundance of Env spike on pseudotyped viruses (~60–70) versus fully infectious viruses (~12–15) (28).

One caveat to the Trx1 supplementation experiments is that we cannot exclude the possibility that enhanced reduction of the CD4 D2 disulfide is facilitating entry. The use of lysis assays in various cell-free contexts has been our method of trying to isolate the mechanism of Trx1/thiol-mediated disulfide exchange to the HIV-1 Env spike alone. In future work, we plan to expand the investigation of redox in HIV-1 infection with CD4-independent Env isolates and CD4 mutants with fixed isomerization states.

### Activating inhibitors transiently induce Env transformation and gp120 shedding

A key common factor between all of the “conformational activator” inhibitors tested here is the ability to induce Env transformation, commonly leading to gp120 shedding from the Env trimer (28, 33, 43, 57, 58). Such destabilization indicates a perturbation of the Env trimer’s metastable state. The results with preincubations (Fig. 4) further support this perturbation hypothesis. The time-limited window observed suggests that Env activation is transient before landing in a local energy minimum embodying inert gp120 and irreversible inactivation of the spike.

In contrast, exposure of virions to Trx1 before KR13b addition reduced the lytic effect, suggesting that the Env spike is being altered from its triggerable, metastable state. Whether these alterations arose by prematurely breaking the C296–C331 bond or if subsequent exchanges also occur is yet unknown, however, and will be part of our next phase of research. A key experiment to bridge the preincubation and infection enhancement experiments will be determining if Trx1-preincubated virus has reduced infectivity, correlating with the reduced lysis observed once KR13b is introduced.

Prior experiments against a panel of HIV-1 Env subtypes across clades and tiers resulted in largely similar lysis profiles, with EC_50_ values ranging from 0.7 to 26 µM and averaged over 70% p24 release (28). Overall, this argues for a conserved mechanistic effect that could broadly impact the breadth of HIV-1 subtypes.

**Fig 7:**
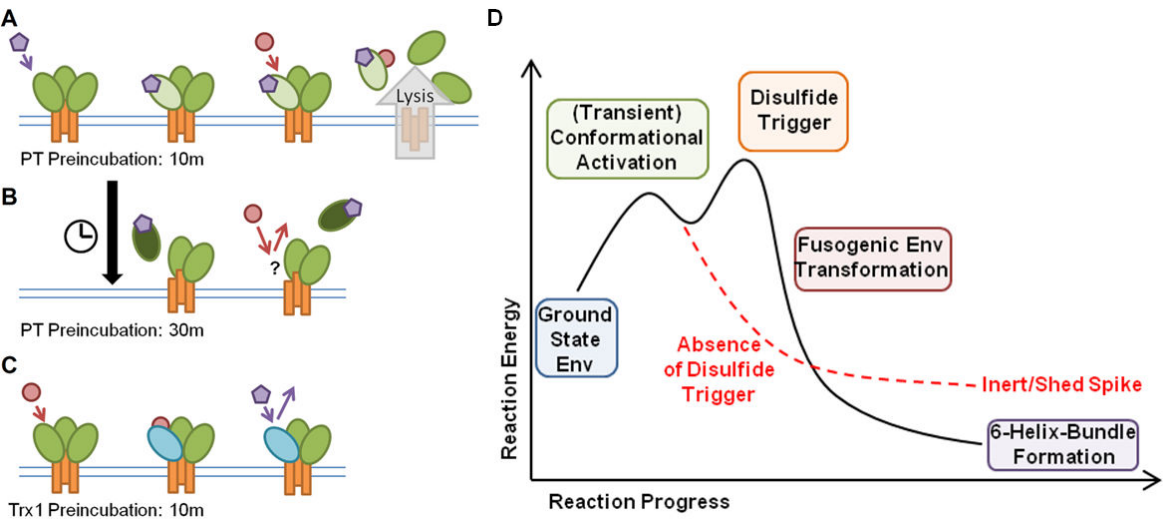
Schematic proposing PT and Trx1 preincubation outcomes. (A) Binding of PT (purple pentagon) to Env causes transient conformational activation of Env. Trx1 (red circle) can then trigger a disulfide exchange in Env, resulting in Env transformation and membrane disruption. See black reaction pathway in panel D. (B) Extended interaction between PT and Env exceeds transient conformational activation and leads to inert form of Env, possibly including gp120 shedding. Trx1 is then unable to trigger disulfide exchange and transformation in the inert Env. See red dashed reaction pathway in panel D. (C) Approach of Trx1 in the absence of conformational activation triggers non-canonical disulfide exchanges, resulting in atypical and inert Env configurations. PT is unable to interact with the atypical configuration Env. (D) Energy diagram depicting reaction path for lytic/fusogenic Env transformation (black line, analogous to A) or alternative non-trigger path (red dashed line, analogous to B). Colored boxes denote key states and events in the lytic/fusogenic Env transformation reaction, unreacted ground-state Env (blue), transient conformational activation of Env into a triggerable state (green), and actual disulfide exchange (orange) triggering the full fusogenic transformation program of Env (dark red), resulting in six-helix bundle formation (purple). Sufficient time spent in the conformational activation (green) without disulfide trigger and may result in the off-path (red dashed line), resulting in an inert and non-infectious Env spike possibly having shed its gp120 subunits.

Based on our observations, we propose that the preincubation with PT is causing a transient activation (Fig. 7A and B), as has been observed with both soluble CD4 and small-molecule CD4 mimetics (42). At the 10-min preincubation window, Env gp120 is activated and can effectively proceed through transformation and lysis if Trx1 is present (Fig. 7A). However, a longer preincubation may allow the PT interaction with Env gp120 to proceed further into an inert gp120-shed state wherein Trx1 has no effect (Fig. 7B). In contrast, preincubation with Trx1 (Fig. 7C) appears to blunt the ability of PT to induce lysis in the complete solution of virus, PT, and Trx1, which may be the result of Trx1 altering disulfide configurations necessary for lysis (but presumably not the result of Trx1 itself inducing premature shedding, as was shown did not occur in Fig. S1). Although C296–C331 is the preferred target of Trx1 (22), without the conformation induced by activation, subsequent exchanges by freed C296 and C331 thiols may occur uncontrolled. Env needs to undergo conformational activation first, entering a transient activated state vulnerable to a disulfide exchange trigger, followed by the actual trigger, leading to transformation (Fig. 7D, black reaction pathway). If no disulfide exchange is triggered during the transient activated state, Env proceeds to become an inert spike as expected from treatment with entry inhibitors (Fig. 7D, red dashed reaction pathway).

Although the preincubation experiments were performed with PT and Trx1, the concept of transient activation is not unknown in the field of small-molecule CD4 mimetics either, as low concentration and/or short interval treatment may result in a brief activation phase of Env before moving into a net inactivation phase (43). If all of the “conformational activator” compounds cause similar rearrangements, transient activation of Env could logically extend to PT, cPT, soluble CD4, and other CD4 mimetics. A secondary observation is that not all of the tested conformational activators are known to interact with Env’s coreceptor site but still resulted in lysis when combined with Trx1, despite HIV-1 generally requiring tropism-dependent coreceptor interaction for fusion. Adding coreceptor signaling could function by stabilizing the transiently activated conformation (analogous to CD4/coreceptor stabilization of Env in State 3 [1, 59, 60]), allowing more time for Trx1 to approach in native infection, perhaps compensated for in our assays with sufficient Trx1 concentration. Future studies could examine if soluble domains of CCR5 or CXCR4 affect co-incubation experiments perhaps by binding to Env during conformational activation and enhancing Trx1 encounter or delaying the decay into inert spike.

### PTT’s thiol serves as a surrogate for native redox enzymes

**Fig 8:**
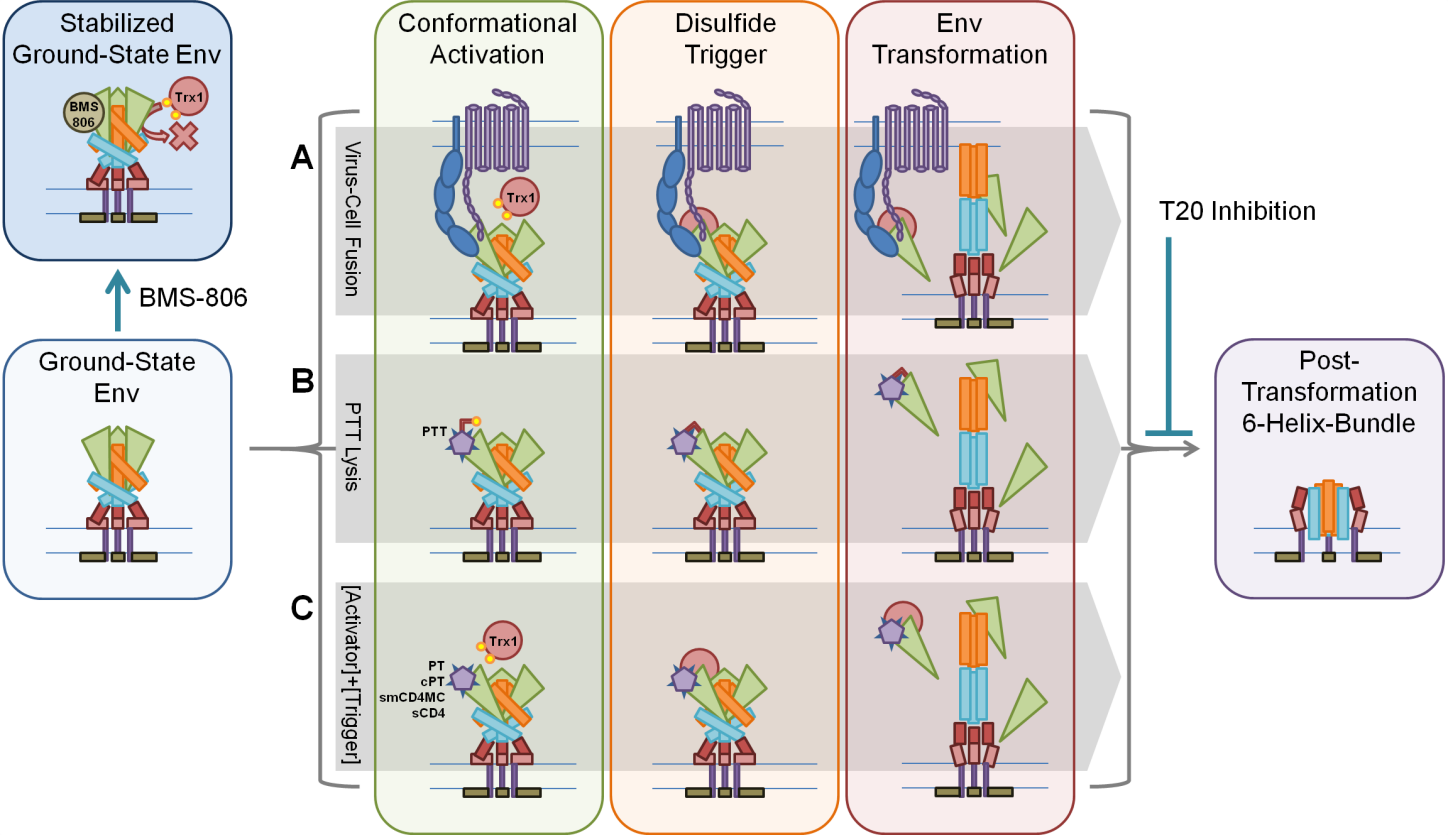
Three pathways taking Env from ground state to post-transformation six-helix bundle. (A) Native fusion mediated by Env interaction with host cell CD4, coreceptor, and Trx1, resulting in virus entry and infection. (B) Transformation mediated by PTT interaction, with IXW pharmacophore acting as surrogate for CD4/CoR inducing conformational activation and free thiol acting as surrogate for endogenous Trx1, resulting in Env transformation, membrane disruption, and content leakage. (C) Conformational activation mediated by Env interaction with various activating components (PT, cPT, small-molecule CD4 mimetics, soluble CD4) and disulfide trigger by exogenous Trx1, resulting in Env transformation, membrane disruption, and content leakage. BMS-806 stabilizes ground-state Env, preventing Trx1-induced transformation. T20 blocks six-helix bundle formation, preventing Trx1-induced transformation, as well.

In recapitulating the lytic effect of the PTT KR13 with a combination of its thiol-blocked analogue KR13b and recombinant Trx1, we have demonstrated that the PTT’s free thiol and exogenous Trx1 can provide the same function in triggering lytic transformations of the Env trimer. Moreover, besides PT compounds, we also demonstrated that cPT, soluble CD4, and small-molecule CD4 mimetics are all capable of activating the Env trimer for Trx1-triggered lytic transformations. This leads us to propose the overall generalized model shown in Fig. 8, delineating multiple conformational activation/disulfide trigger configurations that all transform the ground-state Env through to the six-helix bundle configuration. In addition to the implications about the mechanistic role of the disulfide trigger in enhancing Env transformation, this model suggests using disulfide triggers for Env inactivation and antagonism of HIV infection. Prior work has suggested the potential of doing this by targeting Trx1s and PDIs, but this has significant potential for unintended off-target effects due to the ubiquity of Trx1, PDI, or their analogues (15, 21, 61). In contrast, an Env-binding guide molecule, such as the conformational activators, could bring a thiol or disulfide enzyme close for the premature, cell-free triggering of such an exchange, resulting in irreversible inactivation and potential exposure of key epitopes for immunotherapy.

Our hypothesis on the distinction between fusion and lysis is that, while the same mechanisms are being engaged as native fusion, the critical difference between the two is proximity to the target cells, which were not included in the lysis assays (Fig. 2, 3A and B). Future experiments will investigate incubation of CD4-dependent virus with CD4-negative target cells (with or without coreceptor expression) and attempts to force fusogenic entry by addition of PTTs or [PT + Trx1]. Surrogate cell–cell fusion assays or use of fusion reporter assays could also be used to examine the effect of forcing fusion.

### Ratio of conformational activator and disulfide trigger necessary for transformation

One potentially important observation made in the current work was the order of magnitude improvement in EC_50_ in comparing a fixed 1:1 molar ratio of KR13b and Trx1 across dilutions (Fig. 2, EC_50_ 317 ± 158 nM) vs. using a fixed KR13b concentration (1,000 nM) with serial dilutions of Trx1 (Fig. 3, EC_50_ 63 ± 25 nM). We hypothesize that Env transformation may require more CD4/coreceptor ligand attachment per trimer than disulfide trigger events (e.g., a trimer may require that each protomer has CD4/coreceptor engagement before the occurrence of disulfide exchange trigger becomes likely). This hypothesis further supports the current understanding of Env states 2 (single/partial CD4-bound trimer) and 3 (full CD4-bound trimer + coreceptor) leading to more open/accessible conformations (1, 59, 60), which may be required for disulfide exchange trigger access and susceptibility. This protomer saturation hypothesis could be further investigated by producing pseudovirus with asymmetric trimers, specifically wild type mixed with an S375W mutation, which resists IXW pharmacophore engagement (23, 62). Treatment with fixed Trx1 concentration and serial dilutions of KR13b or other conformational activators could be compared against both fully wild-type and mixed trimers to determine how many pharmacophore engagements are necessary for lysis. From the opposite approach, asymmetric trimers with wild type and C296E/C331K (30) could be evaluated for lysis to determine how many functional C296–C331 disulfides are needed to elicit transformation.

### Flexibility in linear and macrocyclic peptide triazoles for lytic env transformation

While having the exact same pharmacophore, including click-conjugate, there was an approximately 4-fold difference of EC_50_ values observed between the linear KR13b (63 ± 25 nM) and the cyclic 29b (246 ± 97 nM). We propose that inducing Env transformation depends on both initial binding affinity to Env and sufficient flexibility to remain attached to, and not constrain, Env through the process of transforming. One of the advantages of the cPT macrocyclic ring scaffold is increased pharmacophore stability (33). However, a sterically restricted scaffold may also lack the flexibility to adapt to Env as it shifts into more activated states and either prevent such conformational change in Env or dissociate from the changing Env surface. The Env mutant S143N/V255I resists inhibition moderately by cPT 29b and greatly by PTT KR13 without impacting either compound’s binding, suggesting that KR13 may exploit conformational change to a greater degree than 29b as part of its inhibition mechanism (63).

### Disulfide trigger binding and potential for viral inactivation

While we have found that Trx1 can function as a disulfide trigger when in the presence of a conformational activator, experiments substituting cysteine or glutathione as the disulfide trigger source did not cause transformation and lysis (Fig. S4). Trx1 may function as a disulfide trigger because it can bind to Env before initiating disulfide exchange. Of note, a PDI–CD4–CXCR4–gp120 complex has been reported previously based on colocalization in fluorescence microscopy and coimmunoprecipitation (49). Such binding in our assay system needs future evaluation (e.g., by coimmunoprecipitation) for such complex formation with Trx1, as well as other redox enzymes, such as Glutaredoxin, also identified as affecting HIV entry (7, 8). Nonetheless, we know that PTTs can bind to Env via the activator component and therein guide the thiol component to Env disulfides and keep it locally available. Therefore, PTTs and, by extension, macrocyclic peptide triazole thiols can provide a blueprint for future activator–trigger development, including by activator affinity improvement and trigger redox potential.

## Data Availability

The authors confirm that the data supporting the findings of this study are available within the article itself and its supplemental material.

## SUPPLEMENTAL MATERIAL

Supplemental material jvi.01471-24-s0001.docx Figures S1 to S4.

ASM does not own the copyrights to Supplemental Material that may be linked to, or accessed through, an article. The authors have granted ASM a non-exclusive, world-wide license to publish the Supplemental Material files. Please contact the corresponding author directly for reuse.
