# Supplementary material for: Conformational activation and disulfide exchange in HIV-1 Env induce cell-free lytic/fusogenic transformation and enhance infection
Source: J Virol. 2025 Feb 6;99(3):e01471-24. doi: 10.1128/jvi.01471-24 (PMC11915811; doi:10.1128/jvi.01471-24)

**Supplemental Information**

**Supplemental Figure S1. Trx1 addition does not alter PT-induced gp120 shedding.** JRFL Pseudovirus was mixed with serial dilutions of either KR13b+Trx1 in a 1:1 molar ratio, KR13b alone, or Trx1 alone, and incubated for 2 hours at 37°C as in the p24 lysis assays. Supernatants were recovered after centrifugation and loaded onto ELISA plates that were pre-coated with 16H3 anti-gp120 monoclonal antibody for capture and pre-blocked with 3% Bovine Serum Albumin in Phosphate Buffered Saline. Detection was performed using a cocktail of F105/PGT121/PGT145 monoclonal antibodies and an HRP-linked anti-human-IgG secondary antibody. Calculated EC50 values were 68±21 nM for KR13b+Trx1 and 74±21 nM for KR13b alone. Data shown are the mean of three independent experiments, and error bars represent the standard deviation about the mean.


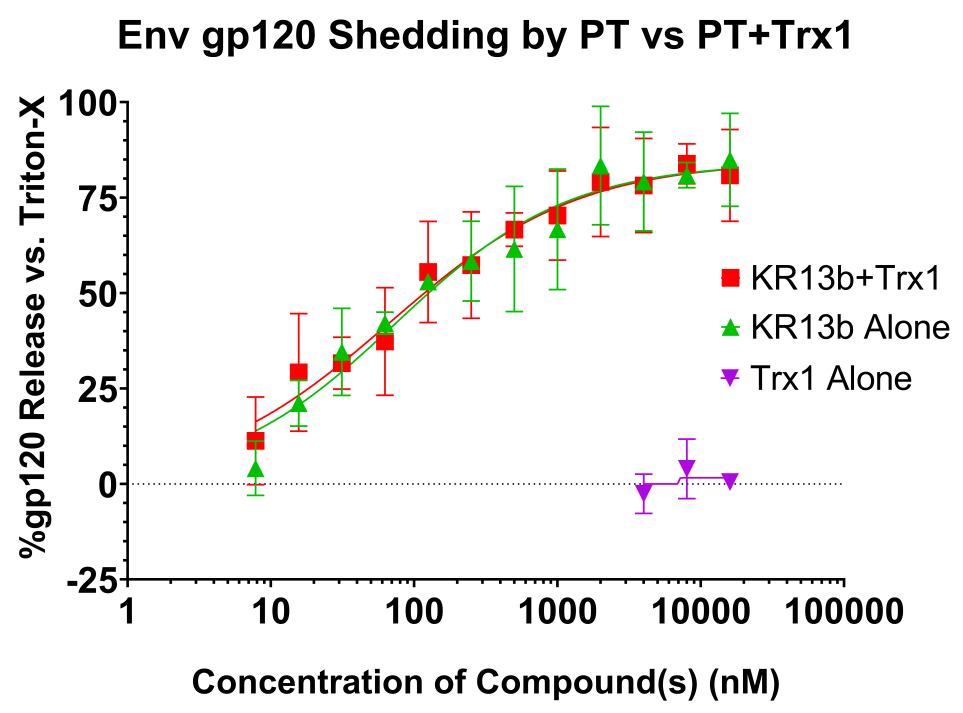


**Supplemental Figure S2. Macrocyclic peptide triazole conformational activators.** Structures are: AAR029b (33), AAR029N2 (34), and MG-II-20 (40).


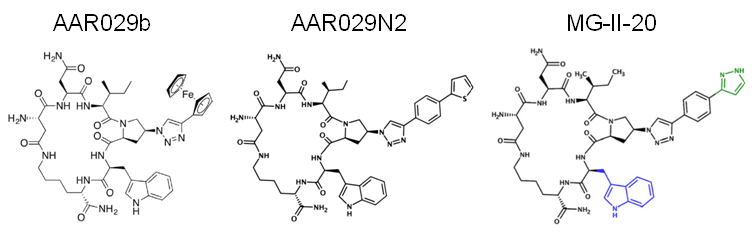


**Supplemental Figure S3. Env-targeting conformational effectors.** Structures are: BNM-III-170 (35), CJF-III-288 (36), and BMS-806 (43).


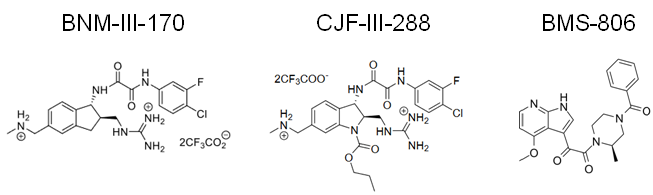


**Supplemental Figure S4. PT KR13b combined with cysteine or glutathione does not enable virus lysis.** JRFL Pseudovirus was mixed with 1000 nM of KR13 alone, KR13b alone, or KR13b plus 1000 nM reduced Cysteine (Cys) or reduced Glutathione (GSH), then incubated for 2 hours at 37°C. Samples were otherwise processed as in the p24 lysis assays. Data shown are the mean of three independent experiments, and error bars represent the standard deviation about the mean.


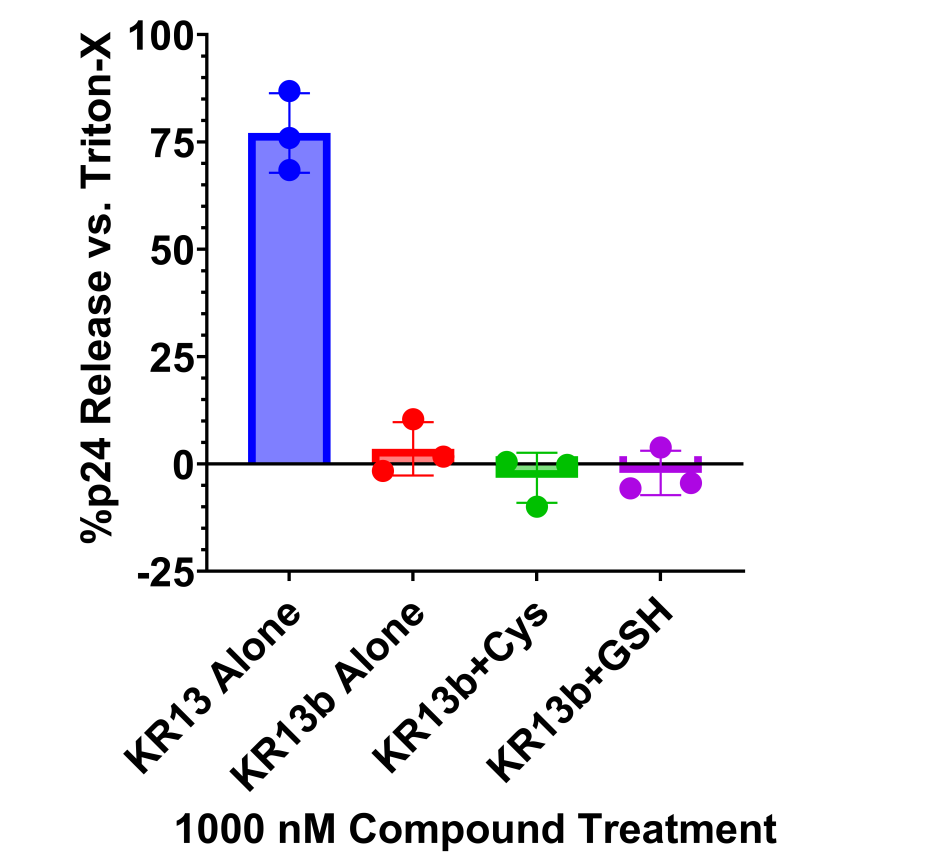

Supplement: Supplemental material — Figures S1 to S4. [file jvi.01471-24-s0001.docx]
